# Association Between Connectivity of Hippocampal Sub-Regions and Auditory Verbal Hallucinations in Schizophrenia

**DOI:** 10.3389/fnins.2019.00424

**Published:** 2019-05-03

**Authors:** Lin Liu, Long-Biao Cui, Yi-Bin Xi, Xing-Rui Wang, Yu-Chen Liu, Zi-Liang Xu, Hua-Ning Wang, Hong Yin, Wei Qin

**Affiliations:** ^1^School of Life Sciences and Technology, Xidian University, Xi’an, China; ^2^Department of Clinical Psychology, School of Medical Psychology, Fourth Military Medical University, Xi’an, China; ^3^Department of Radiology, Xijing Hospital, Fourth Military Medical University, Xi’an, China; ^4^Department of Psychiatry, Xijing Hospital, Fourth Military Medical University, Xi’an, China

**Keywords:** auditory verbal hallucinations, schizophrenia, masked ICA, hippocampus, sub-regions, functional connectivity

## Abstract

**Background:** Hippocampal dysconnectivity has been detected in schizophrenia patients with auditory verbal hallucinations (AVHs). Neuroanatomical evidence has indicated distinct sub-regions in the hippocampus, but which sub-regions within the hippocampus may emerge dysfunction in the brain network, and the relationship between connection strength and the severity of this debilitating disorder have yet to be revealed. Masked independent component analysis (mICA), i.e., ICA restricted to a defined region of interest, can provide insight into observing local functional connectivity in a particular brain region. We aim to map out the sub-regions in the hippocampus with dysconnectivity linked to AVHs in schizophrenia.

**Methods:** In this functional magnetic resonance imaging study of schizophrenia patients with (*n* = 57) and without (*n* = 83) AVHs, and 71 healthy controls, we first examined hippocampal connectivity using mICA, and then the correlation between connection metric and clinical severity was generated.

**Results:** As compared with patients without AVHs, mICA showed a group of hyper-connections for the left middle part, as well as another group of hypo-connections for the bilateral antero-lateral and right antero-medial parts in patients with AVHs. Connectivity was linked to the clinical symptoms scores in the sample of patients with AVHs.

**Conclusion:** These findings demonstrate that the left middle part is more densely connected, but the bilateral antero-lateral and right antero-medial parts are more sparsely connected in schizophrenia patients with AVHs. The findings in the present study show proof of precious location in the hippocampus mediating the neural mechanism behind AVHs in schizophrenia.

## Introduction

Hippocampal abnormalities have been implicated in the pathophysiology of hallucinations in schizophrenia (Behrendt, 2010, 2016). Converging lines of evidence point to an association between the hippocampus and auditory verbal hallucinations (AVHs) in schizophrenia (Jardri et al., 2011; Alderson-Day et al., 2016). Structural findings include a positive correlation between AVHs severity and hippocampal volume principally affecting the right side (Modinos et al., 2009) and a reduction in the total gray matter volume in schizophrenia with AVHs (Maller et al., 2012). Functional abnormalities have been reported in a variety of studies. There is evidence of reduced connectivity between the left hippocampus and superior temporal gyrus and increased connectivity between the left hippocampus/fusiform gyrus and thalamus in patients with AVHs (Sommer et al., 2012; Clos et al., 2014). Disrupted directed thalamic-auditory cortical-hippocampal connectivity has been found to be involved in AVHs in schizophrenia (Li et al., 2017). Studies comparing auditory and visual hallucinations continue to elucidate the contribution of hippocampal connectivity to schizophrenia. In patients with auditory and visual hallucinations, the hippocampus is hyperconnected to the medial prefrontal cortex and caudate, and there is higher white matter connectivity between the hippocampus and visual cortex (Amad et al., 2014), although no difference was detected in hippocampal connectivity between auditory hallucinations and auditory and visual hallucinations (Ford et al., 2015). Moreover, left hippocampal amplitudes of low-frequency fluctuations (ALFF) across all of the cases were related to reported hallucination severity in both auditory and visual domains (Hare et al., 2017). Patients who were experiencing hallucinations (24 out 25 patients had auditory hallucinations) were linked to effective connectivity from the hippocampus to the salience network (Lefebvre et al., 2016). Particularly, integrating ALFF and functional network connectivity, hippocampal ALFF was positively associated with functional network connectivity between the primary auditory cortex and the salience network in patients reporting auditory hallucinations (Hare et al., 2018). From the neuroanatomical perspective, the hippocampus consists of the dentate gyrus, hippocampus proper, and subiculum (Fogwe and Mesfin, 2018). However, it remains unclear which part in the hippocampus with dysconnectivity is linked to this core psychopathology of schizophrenia.

On structural MRI, the hippocampus is segmented into six sub-regions (Dalton et al., 2017). Despite a harmonized protocol for manual segmentation of hippocampal and parahippocampal sub-regions developed by the Hippocampal Subfields Group (Wisse et al., 2017), hippocampal sub-region segmentation is evolving and plays a limited role in fMRI research. To this end, masked independent component analysis (mICA), i.e., ICA restricted to a defined region of interest, is a high-resolution functional parcellation technique and provides insight into observing local functional connectivity in a particular brain region (Blessing et al., 2016; Moher Alsady et al., 2016). Using mICA, subdivisions of the caudate showing reduced cortical functional connectivity have been established in patients with traumatic brain injury (De Simoni et al., 2018).

Given such a background, we aimed to map out the sub-regions with dysconnectivity in the hippocampus associated with AVHs in schizophrenia. We hypothesized that dysconnectivity can be harnessed via mICA to aid in identifying symptom-specific pathophysiology of schizophrenia.

## Materials and Methods

**FIGURE 1:**
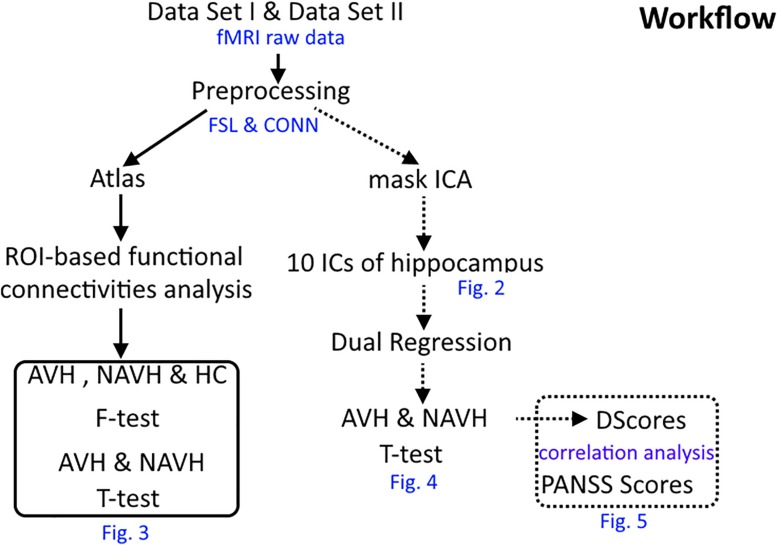
The workflow of this research.

The workflow of this research is presented in Figure 1.

### Participants

Two independent datasets were recruited in this study (Cui et al., 2017c, 2018). Dataset 1 included 74 patients (33 with AVHs, 43 without AVHs) with schizophrenia and 35 healthy controls, which were collected from May 2011 to September 2013 (Cui et al., 2017c). The structural clinical interview for Diagnostic and Statistical Manual of Mental Disorders, Fourth Edition, Text Revision (DSM-IV-TR) was used, and consensus diagnoses were made using all of the available information. Each patient was assessed by using the Positive and Negative Syndrome Scale (PANSS) at the time of imaging. Dataset 2 included 64 patients (24 with AVHs, 40 without AVHs) with schizophrenia and 36 healthy controls, a partial sample of the dataset investigated in Cui et al. (2018). Patients were diagnosed according to DSM, Fifth Edition (DSM-5) from April 2015 to December 2017, with no more than two weeks of cumulative exposure to antipsychotics. We have previously described the criteria for AVHs and Non-AVHs (Chang et al., 2015; Cui et al., 2016, 2017a, 2017b, 2018). This study was approved by the local ethics committee. All participants (or their parents for those under age of 18 years) gave written informed consent after a full description of the aims and design of the study. Table 1 provides further details of the two patient populations.

**TABLE 1:** Clinical and demographical data.

	Dataset 1				Dataset 2			
Characteristic	AVHs (*n* = 33)	Non-AVHs (*n* = 43)	*P* values	HCs (*n* = 35)	AVHs (*n* = 24)	Non-AVHs (*n* = 40)	*P* values	HCs (*n* = 36)
Age (y)	24.7±6.6	25.5±5.6	0.53	27.1±7.3	21.6±5.5	23.7±7.3	0.49	29.3±9.1
Gender (M/F)	17/16	26/17	0.24	22/13	12/12	27/13	0.08	14/22
Education level (y)	13.0±2.0	13.3±1.8	0.41	13.6±3.5	12.4±2.9	12.3±2.9	0.92	15.0±4.1
Duration of illness (mon)	23.0±26.7	20.4±29.2	0.80	NA	8.2±13.5	15.4±20.6	0.12	NA
PANSS score								
Total score	100.2±18.5	91.1±15.1	0.06	NA	85.2±16.7	85.0±15.1	0.52	NA
Positive score	27.5±7.8	21.2±5.3	0.33	NA	23.5±5.3	20.8±5.8	0.80	NA
Negative score	25.5±7.1	21.2±7.8	0.15	NA	17.1±7.0	20.9±7.7	0.44	NA
General psychopathology score	47.2±9.5	48.7±8.6	0.65	NA	43.6±8.8	43.3±8.3	0.51	NA

*HCs, healthy controls; PANSS, Positive and Negative Syndrome Scale.*

### Image Acquisition

High-resolution structural imaging and resting-state functional data were acquired on a Siemens 3.0 T scanner for dataset 1 and a GE 3.0 T scanner for dataset 2 using protocols published previously (Cui et al., 2019). More details are shown in Supplementary Table 1. Those participants whose head motion exceeded more than 2.5 mm or 3.0° during resting-state functional MRI were detected and removed using our own Matlab scripts.

### Data Preprocessing

Functional imaging data were processed using tools from CONN toolbox and FSL 5.0.11 (Oxford Centre for Functional MRI of the Brain, Oxford, United Kingdom^1^), mICA^2^ and scripts written in Matlab (MathWorks, Natick, MA, United States). Anatomical images were segmented using FEAT of FSL into gray matter, white matter and cerebrospinal fluid (CSF) using FEAT. Preprocessed functional data consisted of 240 time points, and due to a decrease in the magnetic field effect the first 10 volumes were removed. Preprocessing included realignment of echo-planar images to remove the effects of motion (using Motion Correction FMRIB’s linear image registration tool, MCFLIRT), brain extraction (using Brain Extraction Tool, BET), spatial smoothing with a 6 mm full width at half maximum Gaussian kernel, and high-pass temporal filtering by using a cutoff frequency of 150 sec (∼0.007 Hz). The Motion Correction FMRIB’s non-linear image registration tool (FNIRT) was used to register functional MR imaging volumes into standard montreal neurological institute (MNI) space templates (step1: individual functional space to individual structural space; step2: individual structural space to MNI standard space), and post-registration images were re-sampled to 2 mm for further analysis (Cui et al., 2017b, 2018). Furthermore, the factors of sex and age of all subjects were regressed using the FSL toolbox. In order to avoid the impact of the two different datasets in imaging, we compared the data preprocessing from the two datasets and did not find any significant differences (Supplementary Figure 1 and Supplementary Table 2).

### mICA: Based on Hippocampus

**FIGURE 2:**
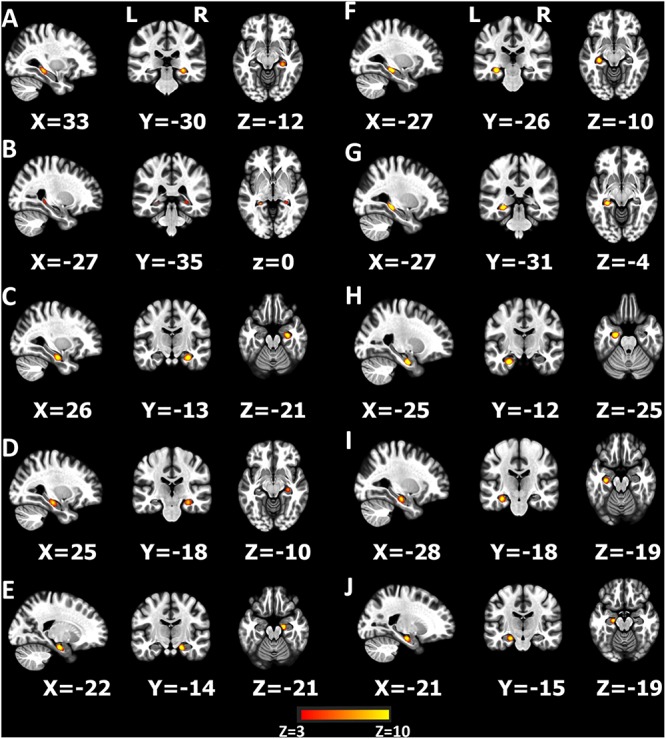
Ten components produced by independent component analysis (ICA) restricted within the hippocampus **(A–J)**. The *z*-scores > 3 are colored in the figure.

Further analysis after preprocessing was implemented in mICA tools (see footnote 2) for both patients and healthy controls. Based on Blessing et al.’s (2016) research, left and right hippocampi in the Harvard-Oxford subcortical structural atlas were selected as the seed ROIs, and dimensionality of 10 was selected as the number of components (Figure 2; Blessing et al., 2016; Moher Alsady et al., 2016). In order to verify the stability of the ingredients, the 10 independent components (ICs) were identified in all subjects of two data sets separately (GE data set and Siemens data set) as suggested by Blessing et al. (2016) and then we compared the spatial correlation of each of the 10 ICs (Supplementary Figure 1 and Supplementary Table 2). The 10 hippocampus components generated by the combined data set were selected as the ICs for further analysis.

### Functional Connectivity Analyses Based on 10 Hippocampus Components

Multivariate functional connectivity between 10 components and the whole brain was assessed via a modified dual regression approach. Data analysis was implemented in FSL tools (see footnote 1) according to the method of Filippini et al. (2009). Briefly, in the first step of dual regression, the concatenated multiple functional MR imaging data sets were decomposed by using ICA, in which 10 hippocampus components were applied to detect large-scale patterns of functional connectivity in subjects. Second, the dual-regression approach was used to identify subject-specific temporal dynamics and associated spatial maps within each subject’s functional MR imaging data set. This involved using the spatial maps of the group ICA in a linear model fit (spatial regression) against the separate functional MR imaging data sets, resulting in matrices that describe the temporal dynamics of each component and subject. Then, subject-specific spatial maps were estimated by using these time-course matrices in a linear model fit (temporal regression) against the associated functional MR imaging data sets. Third, the different component maps were collected across subjects into single four-dimensional files (one per original ICA map, with the fourth dimension being subject identification) and them tested voxel-wise for statistically significant differences between groups by using non-parametric permutation testing (5000 permutations). The maps were thresholded by using an alternative hypothesis test based on a mixed Gaussian and gamma distribution model in previous work and by controlling the local false discovery rate at *P* less than 0.05 (Cui et al., 2017b).

### ROI-Based Functional Connectivity of the Hippocampus

After preprocessing, ROI-based correlational analyses were performed using the Functional Connectivity (CONN) toolbox^3^ between the hippocampus and whole brain ROIs. Briefly, (1) after preprocessing, the confounding effects of white matter, CSF, realignment and scrubbing were removed using linear regression. Then, (2) linear detrending. (3) first-level analysis, left- and right- hippocampi were selected as the seed ROIs to analyze the functional connectivity between the seed ROIs and all brain ROIs. (4) second-level analysis. Any differences between seed ROIs and all brain ROIs for the group comparison among AVHs, N-AVHs, and HCs using a general linear model (GLM) and differences between AVHs and Non-AVHs were calculated (Cui et al., 2018).

### Statistical Analysis

The difference scores (removed the value > |Mean ± 3SD|) of significant different regions (difference scores, $d\,s\,c\,o\,r\,e=\left|\sum_{R=1}^{k}\sum_{i=1}^{n}S_{i,R}\right|$, *R*, the difference regions of each hippocampus component; *k*, the number of regions; *S*, the scores of difference regions; and *i*, the number of voxels of each significant different region) were calculated for AVHs and Non-AVHs patients. The correlation between PANSS scores and difference scores was calculated for patients.

## Results

### Clinical Characteristics

Table 1 shows the full description of demographic and clinical characteristics of patients and healthy controls. No significant difference was found in age, gender, education, and handedness among the patient groups.

### mICA Findings-Based Connectivity

**FIGURE 3:**
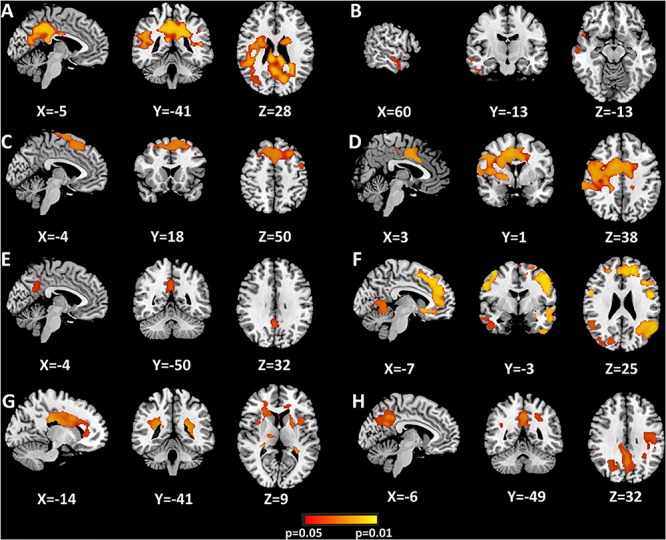
Significant differences of hippocampus components between AVHs and Non-AVHs using dual regression **(A–H)**. Correction via Bonferroni and the areas where *P* < 0.05 are shown in the figure. The color bar indicates the *P-*value.

Figure 3, Table 2, and Supplementary Table 3 summarize the regions where hippocampal components-seeded connections were altered between AVHs and Non-AVHs. Supplementary Figure 2 and Table 3 show the mean connection strength in each group. In contrast to patients without AVHs, patients with AVHs had increased connection strength in the left middle part and decreased connection strength in the bilateral antero-lateral and right antero-medial parts.

**TABLE 2:** The differences areas of mICA.

Position	Number of voxels	Peak MNI coordinate			Peak intensity
		x	y	z	
Anterior.L	6578	−26	−54	28	0.99
Anterior.R	362	52	−14	−32	0.966
Antero-medial.L	5751	0	18	50	0.976
Antero-medial.R	8944	40	−18	−18	0.986
Antero-lateral.L	593	4	−58	24	0.966
Antero-lateral.R	32601	−40	32	−20	0.992
Middle.L	2743	−16	−26	26	0.984
Middle.R	3512	2	−56	24	0.974

**TABLE 3:** Mean connection strength for each group.

	AVHs		NAVHs		HCs		AVHs vs. HCs	AVHs vs. NAVHs	NAVHs vs. HCs
	mean	Std	mean	std	mean	std			
Antero-lateral.L	4.64	1.57	5.75	1.68	4.44	1.39	*p* = 0.55	*p = 0.04	*p = 0.03
Antero-medial.L	3.76	1.79	4.23	1.83	3.29	1.64	*p = 0.03	*p* = 0.32	*p = 0.04
Posterior.L	4.54	1.67	4.89	1.81	4.30	1.49	*p = 0.04	*p* = 0.57	*p = 0.04
Middle.L	11.81	2.09	6.60	2.00	5.73	1.71	**p = 0.00	**p = 0.00	*p* = 0.73
Middle.R	2.53	1.61	2.06	1.40	3.73	1.80	**p = 0.01	*p* = 0.95	**p = 0.01
Antero-lateral.R	6.19	1.93	7.47	2.03	5.86	1.61	*p* = 0.48	*p = 0.02	*p = 0.01
Posterior.R	5.30	1.90	5.81	1.94	4.99	1.64	*p* = 0.85	*p* = 0.43	*p = 0.03
Antero-medial.R	3.84	1.50	4.99	1.79	3.64	1.29	*p = 0.03	*p = 0.02	*p = 0.01

*p < 0.05; ^∗∗^*p* < 0.01.

### ROI-Based Connectivity

**FIGURE 4:**
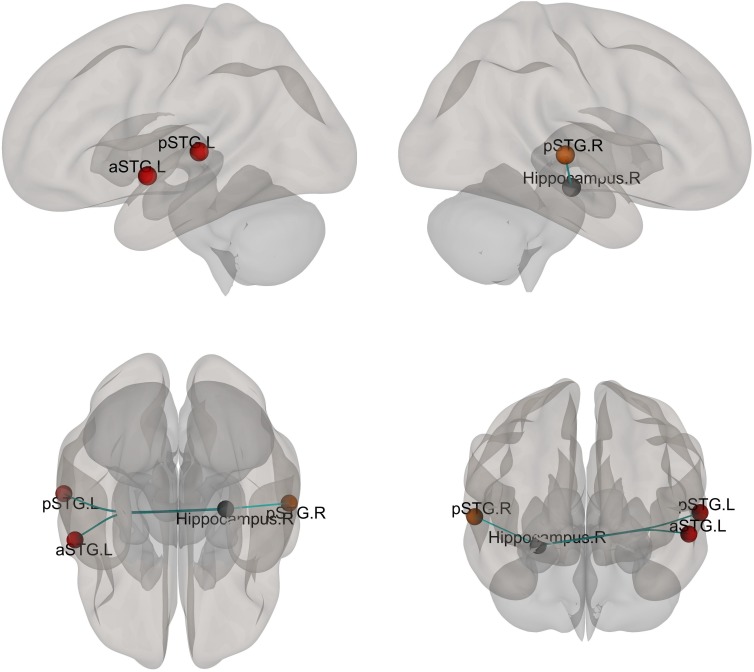
Different functional connectivity between whole hippocampus and ROIs of the brain mask. *F*-tests were calculated and the functional connectivity with a significant difference after FDR correction is shown. The color of the ball indicated the relationship between mask ROI (Hippocampus) and the other ROIs (Gray ball: the mask ROI, the warm color indicated that the connection increased).

The bilateral hippocampi were utilized as seeds for the ROI-based connectivity analysis. A few regions had altered functional connectivity with the seed areas (Figure 4 and Supplementary Table 4). Altered connectivity was seen between the right hippocampus and bilateral superior temporal gyrus among the three groups. No significant difference was found between patients with and without AVHs.

### Clinical Correlates

**FIGURE 5:**
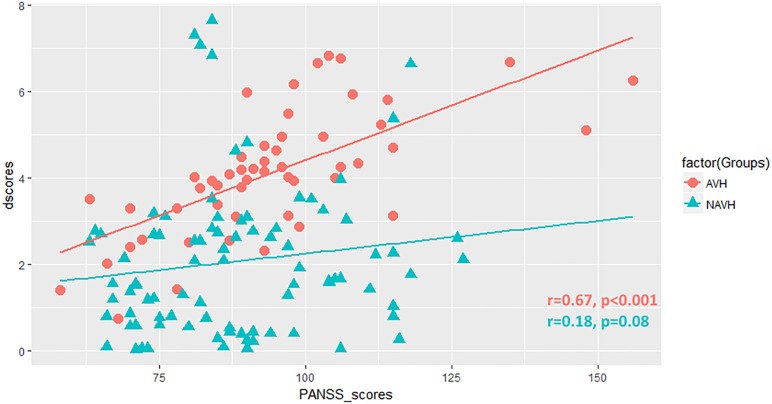
Scatter plot showing the correlation between difference scores (dscores) and PANSS total scores (PANSS_scores) in patients.

Positive correlation was found between difference scores of functional connectivity and PANSS total scores in patients with AVHs (*r* = 0.67, *P* < 0.001), suggesting a significant effect of AVHs on clinical severity (Figure 5). There was no clear association between connectivity metrics and PANSS total scores in patients without AVHs (*r* = 0.18, *P* = 0.08). We also added supplementary analysis to calculate the correlation between PANSS positive, negative, general psychopathology, and supplemental scores and neuroimaging measures (Supplementary Table 5).

## Discussion

Identifying the disrupted hippocampal structure and function in schizophrenia at a sub-large-scale level has a critical role in fundamental research. In this study, combining neuroimaging and mICA analysis, we reported and presented a full view of hippocampal regional connectivity pattern for AVHs in schizophrenia, including bilateral anterior, antero-medial, antero-lateral, middle, and posterior parts.

The present study extends the previous findings (Sommer et al., 2012; Clos et al., 2014; Li et al., 2017) by providing a hippocampal sub-region-level pattern of AVHs-specific disrupted connectivity in schizophrenia via mICA. The unique advantage of mICA is separating the hippocampus into several sub-regions for the connectivity analysis. We explored the sub-regional aspects of hippocampal connectivity in schizophrenia patients with AVHs, which allows and ensures displaying dysfunctional brain regions involving AVHs in schizophrenia. We found patients with AVHs with increased connection strength in the left middle part and decreased connection strength in the bilateral antero-lateral and right antero-medial parts compared with patients without AVHs, indicating disrupted sensory representation or memory underlying hallucinations in this disorder.

In our study, another result is that patients in this cohort showed right hippocampus-seeded dysconnectivity with bilateral superior temporal gyri, which is in line with a previous report (Sommer et al., 2012). With the exception of the superior temporal gyrus, which proved to be a reasonable AVHs-related brain area (Orlov et al., 2018; Spray et al., 2018), we uncovered a much more complicated network for AVHs in schizophrenia by means of mICA. This finding is suggestive of aberrant patterns of multiple brain areas in schizophrenia patients suffering from AVHs, and has made an unexpected discovery of AVH proneness. ROI-based connectivity analysis of the whole hippocampus could be an impediment to detecting some subtle alterations for functional neuroimaging. On the contrary, mICA might be an alternative approach to explore much more changes missed by ROI-based analysis.

Affected memory to some extent is known to exist in cognitive symptoms of schizophrenia (Ricarte et al., 2017). The implications of hallucinatory experience as aberrant event memory formation for the pathophysiology of schizophrenia

have been established (Behrendt, 2016). A sizable number of studies have implicated hippocampal association with AVHs in schizophrenia. A recently proposed model for AVHs in schizophrenia is that “the aberrant neuromodulation within RSNs involving sensory information filtering, auditory processing/language processing, and memory retrieving might implicate a contribution to this distressing symptom” [see Figure 5 in Cui et al. (2017b) for review]. While it remains to be determined, neural mechanisms underlying disrupted connectivity of hippocampal sub-regions, the evidence from this study could support such a model involving memory retrieval.

Several aspects of the current study deserve further comments. With the mICA Toolbox, ICA dimensionality was set to a value of 10 for the hippocampus in our study, but this value was 12 in a previous study (Moher Alsady et al., 2016). The number of components varies from data to data in the ICA decomposition. In addition to 10 hippocampal components, we performed 8, 12, and 15 components, revealing consistent results (for details see Supplementary Materials). The neurobiological underpinnings behind findings of this study are currently not known. Schizophrenia-patient-derivedCA3 neurons from human pluripotent stem cells have been efficiently generated and enable modeling of deficit hippocampal connectivity *in vitro* (Sarkar et al., 2018). What should be done in the future includes linking the network of hippocampal sub-regions in relation to AVHs in schizophrenia using fMRI and cell physiology.

In summary, our study shows that schizophrenia patients with AVHs exhibit disrupted connectivity of hippocampal sub-regions, which represents a core symptom-specific pathophysiology of schizophrenia. Mapping out the sub-regions with dysconnectivity in the hippocampus associated with AVHs in schizophrenia may help elucidate neurobiological substrates of this disorder.

## Ethics Statement

This study was carried out in accordance with the recommendations of the Institutional Ethics Committee, First Hospital of the Fourth Military Medical University with written informed consent from all subjects. All subjects gave written informed consent in accordance with the Declaration of Helsinki. The protocol was approved by the Institutional Ethics Committee, First Hospital of the Fourth Military Medical University.

## Author Contributions

Y-BX, HY, and WQ were responsible for the entire study. All authors contributed to the study concepts and study design, data acquisition, data analysis and interpretation, manuscript drafting, manuscript revision for important intellectual content, approval of the final version of the manuscript, and agreed to ensure that any questions related to the work are appropriately resolved. L-BC, X-RW, Y-BX, and HY contributed to the literature research. LL, L-BC, X-RW, H-NW, and HY performed the clinical studies. L-BC, Y-BX, X-RW, and HY performed the experimental studies. LL, L-BC, Y-CL, Z-LX, HY, and WQ contributed to the statistical analysis. LL, L-BC, HY, and WQ were responsible for the manuscript revision.

## Conflict of Interest Statement

The authors declare that the research was conducted in the absence of any commercial or financial relationships that could be construed as a potential conflict of interest.

## Footnotes

1 http://www.fmrib.ox.ac.uk/fsl/

2 https://www.nitrc.org/projects/mica

3 http://web.mit.edu/swg/software.htm
