# Supplementary material for: Association Between Connectivity of Hippocampal Sub-Regions and Auditory Verbal Hallucinations in Schizophrenia
Source: Front Neurosci. 2019 May 3;13:424. doi: 10.3389/fnins.2019.00424 (PMC6509236; doi:10.3389/fnins.2019.00424)
Supplement: Supplementary file 1 [file Data_Sheet_1.docx]

## Supplementary Table 1. Detailed scanning parameters

|  | **Siemens scanner** | |  | **GE scanner** | |
| --- | --- | --- | --- | --- | --- |
|  | **T1** | **fMRI** |  | **T1** | **fMRI** |
| TR (ms) | 2530 | 2000 |  | 8.2 | 2000 |
| TE (ms) | 3.5 | 30 |  | 3.2 | 30 |
| Flip angle (°) | 7 | 90 |  | 12 | 90 |
| FOV (mm^2^) | 256 × 256 | 220 × 220 |  | 256 × 256 | 240 × 240 |
| Matrix | 256 × 256 | 64 × 64 |  | 256 × 256 | 64 × 64 |
| Slice thickness (mm) | 1 | 4 |  | 1 | 3.5 |
| Section gap (mm) | 0 | 0.6 |  | 0 | 0 |
| Number of slices | 192 | 33 |  | 196 | 45 |

FOV, field of view; TE, echo time; TR, repetition time (ms).

## Supplementary Table 2. Comparison of the data from two datasets

|  | GE data set vs Siemens data set | | |
| --- | --- | --- | --- |
|  | AVHs | NAVHs | HCs |
| Mean | p = .82 | p = .66 | p = .74 |
| Antero-lateral.L | p = .92 | p = .55 | p = .83 |
| Antero-medial.L | p = .77 | p = .68 | p = .75 |
| Posterior.L | p = .63 | p = .73 | p = .79 |
| Middle.L | p = .82 | p = .80 | p = .63 |
| Middle.R | p = .85 | p = .66 | p = .90 |
| Antero-lateral.R | p = .81 | p = .53 | p = .82 |
| Posterior.R | p = .83 | p = .48 | p = .71 |
| Antero-medial.R | p = .85 | p = .89 | p = .52 |

## Supplementary Table 3. Regions where hippocampal components-seeded connections were altered between AVHs and Non-AVHs.

| Coordinates of components | Cluster | Voxels | Peak MNI coordinate | | | Peak intensity | Structure (aal) |
| --- | --- | --- | --- | --- | --- | --- | --- |
|  |  |  | X | Y | Z |  |  |
| -5 -41 28 | 1 | 13855 | -26 | -54 | 28 | 0.99 | 1063 Precuneus_R  917 Precuneus_L  860 Cingulum_Mid_L  719 Cingulum_Mid_R  328 Cingulum_Post_L  319 SupraMarginal_R  306 Temporal_Sup_R  302 Calcarine_L  294 Cuneus_L  236 Cingulum_Post_R  226 Temporal_Mid_R  206 Thalamus_R  188 Temporal_Mid_L  169 Occipital_Sup_L  159 Occipital_Mid_R  144 Angular_R  142 Caudate_L  117 Caudate_R  104 Angular_L  97 Rolandic_Oper_R  93 Parietal_Inf_L  87 Occipital_Mid_L  81 Paracentral_Lobule_R  56 Temporal_Sup_L  54 Frontal_Inf_Oper_R  50 Supp_Motor_Area_R |
| 60 -13 -13 | 1 | 275 | 52 | -14 | -32 | 0.966 | 172 Temporal_Mid_R  92 Temporal_Inf_R |
|  | 2 | 114 | 52 | 12 | -18 | 0.962 | 98 Temporal_Pole_Sup_R |
| -4 18 50 | 1 | 63 | 10 | 42 | 40 | 0.956 | 53 Frontal_Sup_Medial_R |
|  | 2 | 5841 | 0 | 18 | 50 | 0.976 | 940 Supp_Motor_Area_L  677 Frontal_Sup_L  647 Supp_Motor_Area_R  573 Precentral_L  499 Precentral_R  419 Frontal_Mid_L  392 Frontal_Sup_R  385 Postcentral_L  228 Paracentral_Lobule_L  174 Paracentral_Lobule_R  159 Frontal_Sup_Medial_L  154 Postcentral_R  151 Frontal_Sup_Medial_R  131 Frontal_Mid_R  110 Parietal_Inf_L  59 Parietal_Sup_L |
| 3 1 38 | 1 | 16174 | 40 | -18 | -18 | 0.986 | 1002 Rolandic_Oper_R  847 Cingulum_Mid_R  780 SupraMarginal_R  664 Cingulum_Mid_L  661 Precentral_R  554 Postcentral_R  500 Frontal_Inf_Tri_R  489 Frontal_Inf_Oper_R  485 Putamen_R  441 Insula_R  395 Frontal_Mid_R  377 Hippocampus_R  317 Supp_Motor_Area_R  241 Temporal_Sup_R  224 Supp_Motor_Area_L  217 Thalamus_R  216 Pallidum_R  153 Caudate_R  151 Cingulum_Ant_R  103 Heschl_R  67 Parietal_Inf_R  60 Cingulum_Ant_L |
| -4 -50 32 | 1 | 627 | 4 | -58 | 24 | 0.966 | 206 Precuneus_R  197 Precuneus_L  72 Cingulum_Post_L  60 Cingulum_Post_R  58 Cingulum_Mid_R |
| -7 -3 25 | 1 | 35725 | -40 | 32 | -20 | 0.992 | 3070 Temporal_Mid_L (aal)  2208 Precentral_L (aal)  1748 Frontal_Mid_L (aal)  1263 Frontal_Sup_L (aal)  1161 Precentral_R (aal)  1131 Frontal_Mid_R (aal)  1102 Frontal_Sup_Medial_L (aal)  1067 Frontal_Inf_Orb_L (aal)  1062 Frontal_Inf_Tri_L (aal)  957 Parietal_Inf_L (aal)  903 Postcentral_R (aal)  825 Temporal_Sup_L (aal)  781 Supramarginal Gyrus  741 Temporal_Mid_R (aal)  676 Frontal_Sup_R (aal)  650 Cingulum_Ant_L (aal)  618 Occipital_Mid_R (aal)  608 Postcentral_L (aal)  579 Frontal_Inf_Orb_R (aal)  554 Occipital_Mid_L (aal)  538 Calcarine_R (aal)  533 Lingual_R (aal)  497 Parietal_Sup_R (aal)  497 Angular_R (aal)  490 Frontal_Sup_Medial_R (aal)  486 Angular_L (aal)  470 Supp_Motor_Area_R (aal)  431 Supp_Motor_Area_L (aal)  377 Insula_L (aal)  371 Cingulum_Ant_R (aal)  347 Calcarine_L (aal)  333 Temporal_Inf_L (aal)  325 Parietal_Inf_R (aal)  320 Temporal_Pole_Sup_L (aal)  316 Frontal_Inf_Oper_L (aal)  306 Lingual_L (aal)  297 Frontal_Med_Orb_R (aal)  279 Frontal_Inf_Tri_R (aal)  237 SupraMarginal_L (aal)  210 Insula_R (aal)  204 Frontal_Mid_Orb_R (aal)  194 Cerebelum_4_5_L (aal)  182 Frontal_Mid_Orb_L (aal)  171 Putamen_L (aal)  160 Vermis_4_5 (aal)  158 Cuneus_R (aal)  155 Occipital_Sup_R (aal)  153 Caudate_L  152 Frontal_Med_Orb_L |
|  | 2 | 1776 | 66 | -22 | -6 | 0.966 | 927 Temporal_Mid_R  349 Temporal_Sup_R  297 Temporal_Inf_R  135 Temporal_Pole_Mid_R |
|  | 3 | 278 | -26 | -56 | 64 | 0.96 | 216 Parietal_Sup_L  62 Precuneus_L |
|  | 4 | 56 | -2 | -8 | 68 | 0.952 | 54 Supp_Motor_Area_L |
|  |  |  |  |  |  |  |  |
|  |  |  |  |  |  |  |  |
|  |  |  |  |  |  |  |  |

## Supplementary Table 4. Comparison of functional connectivity between whole hippocampus and ROIs of brain mask

| Region | Result of ROI-based FC F-test | Intensity | Size* |
| --- | --- | --- | --- |
| Seed Hippocampus r | X(66) = 105.06 0.0016 0.0016 | 28.63 | 3 |
| Hippocampus r-pSTG l | F(2)(161) = 10.26 0.0001 0.0088 |  |  |
| Hippocampus r-pSTG r | F(2)(161) = 9.67 0.0001 0.0088 |  |  |
| Hippocampus r-aSTG l | F(2)(161) = 8.69 0.0003 0.0142 |  |  |

*Number of connections with significant difference.

## Supplementary Table 5. Correlation analysis between PANSS scores and dscores

|  | **P** | **N** | **G** | **S** | **T** |
| --- | --- | --- | --- | --- | --- |
| AVH-dscores | r = 0.4564 p < 0.001 | r = 0.4603 p < 0.001 | r = 0.5224 p < 0.001 | r = 0.3127 p =0.0179 | r = 0.6705 p < 0.001 |
| NAVH-dscores | r = 0.2687 p = 0.014 | r = -0.0224 p = 0.8405 | r = 0.0405 p = 0.7165 | r = 0.1454 p = 0.1897 | r = 0.1825 p = 0.08 |

P, positive score; N, negative score, G, general psychopathology score; S, supplementary score; T, total score.

## Supplementary figure legends


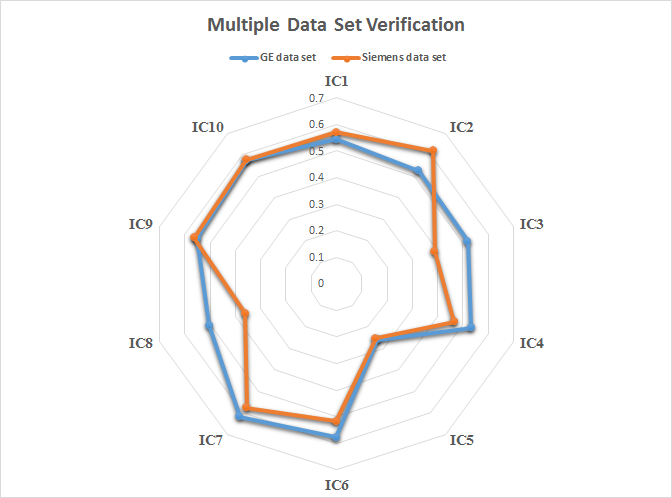


Supplementary Figure 1. The spatial correction among GE data set, Siemens data set and multiple data set to verify the stability of 10 ICs of hippocampus using mICA.


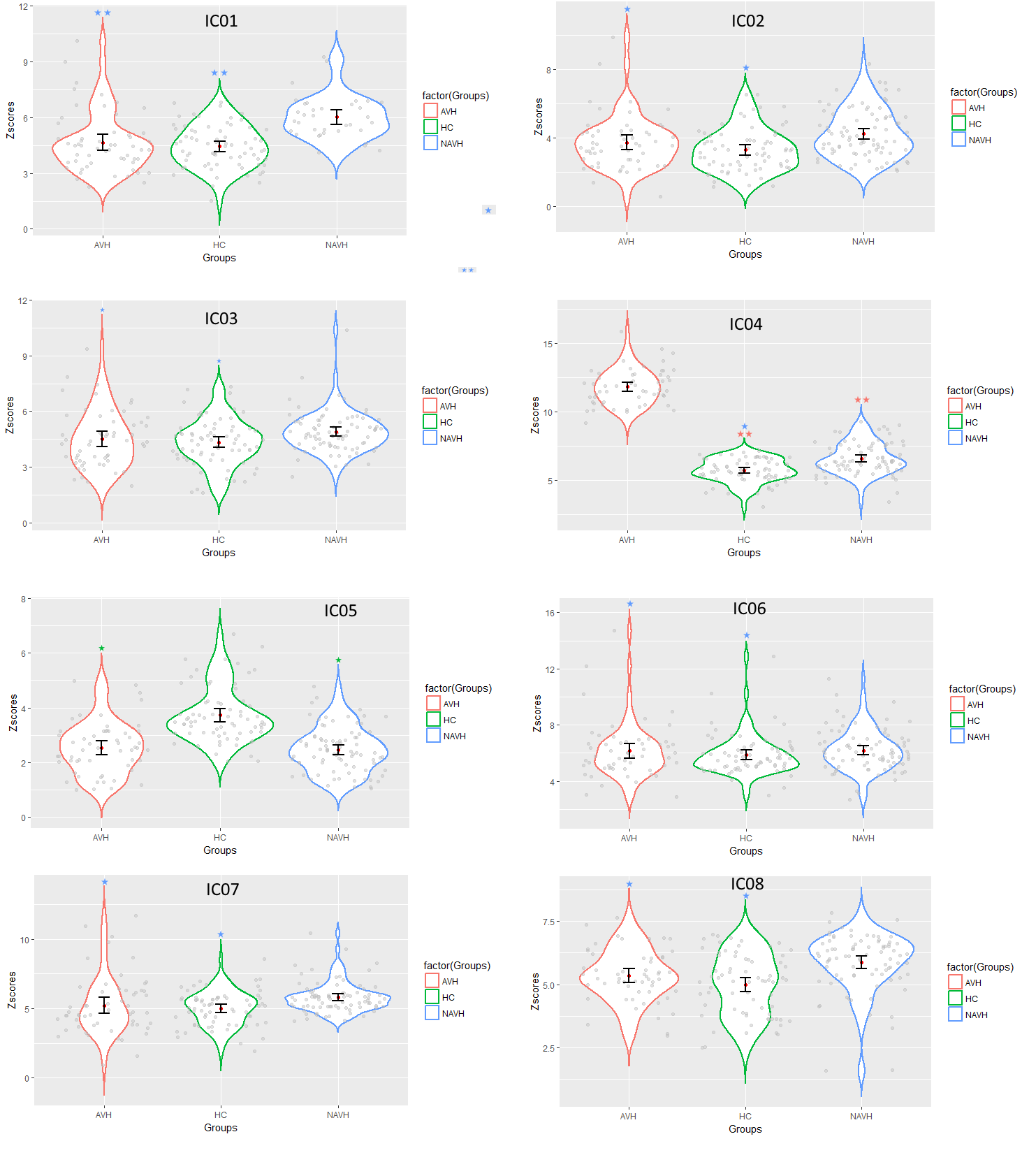


Supplementary Figure 2. Between-group differences of mean connection strength. ^*^*P* < 0.05 versus patients with AVHs (in red); ^**^*P* < 0.01 versus patients with AVHs (in red); ^*^*P* < 0.05 versus patients without AVHs (in blue); ^**^*P* < 0.01 versus patients without AVHs (in blue).


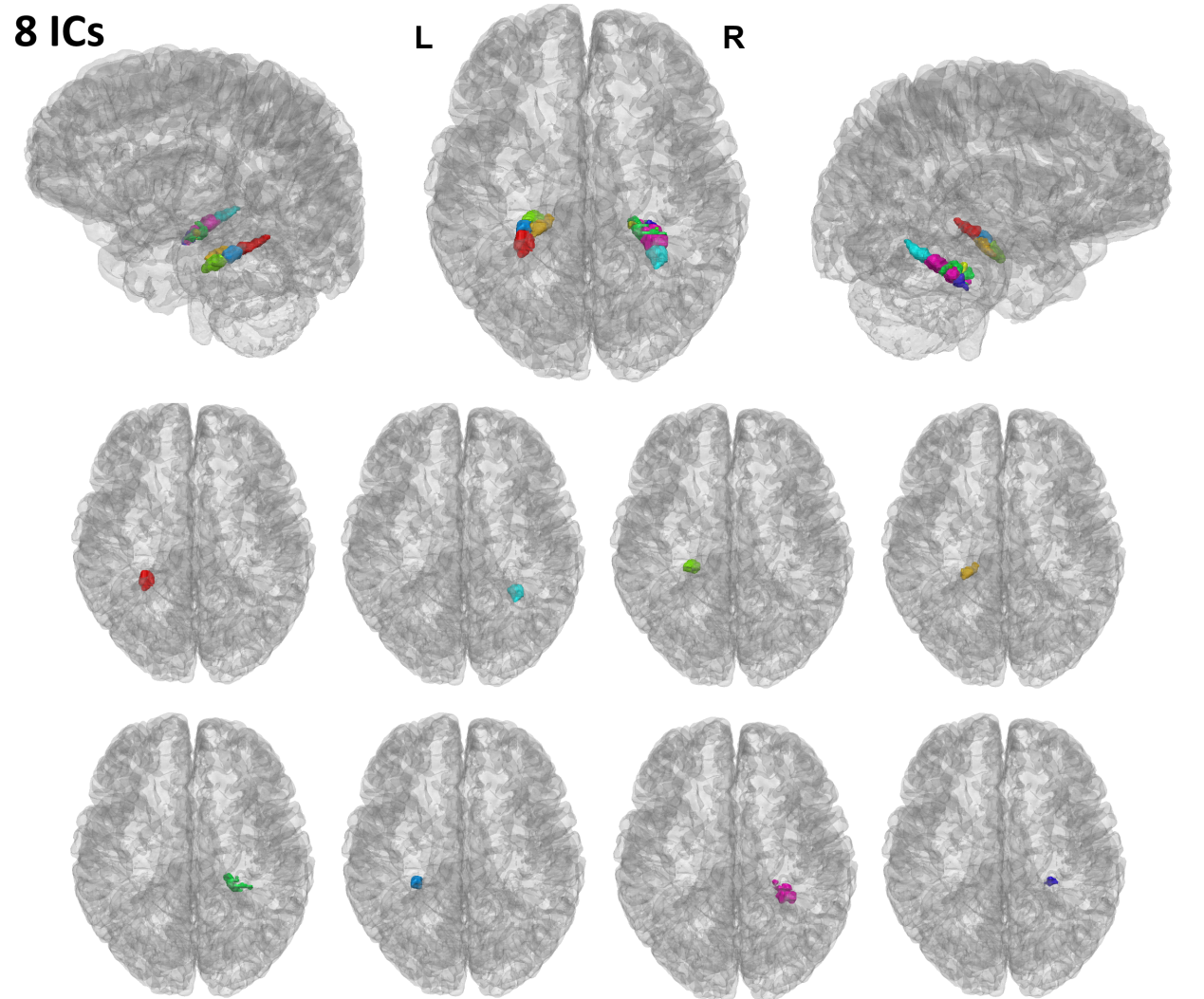


Supplementary Figure 3. Eight components produced by independent component analysis restricted within the hippocampus.


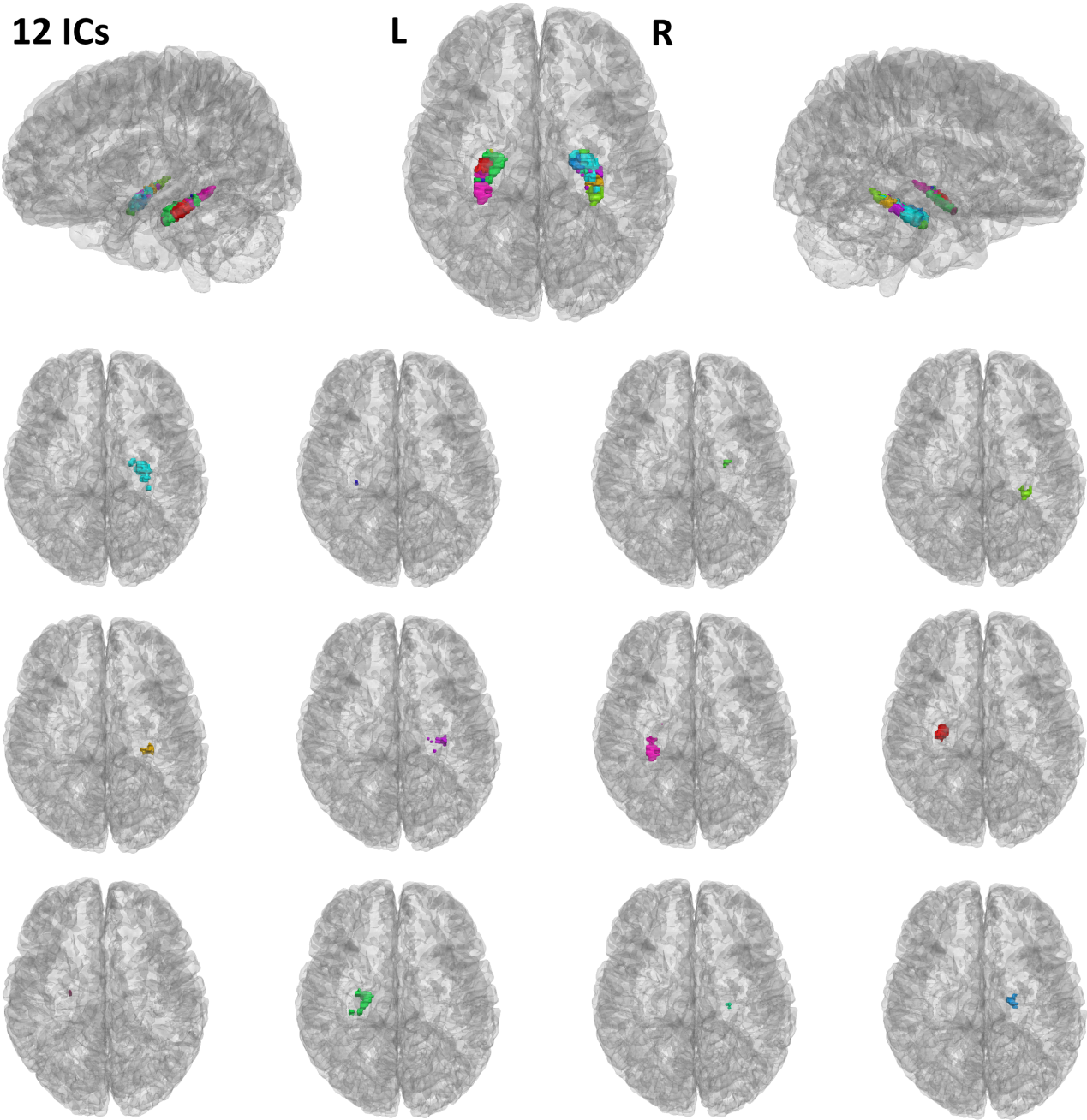


Supplementary Figure 4. Twelve components produced by independent component analysis restricted within the hippocampus.
